# Normative Data of the Wingate Anaerobic Test in 1 Year Age Groups of Male Soccer Players

**DOI:** 10.3389/fphys.2018.01619

**Published:** 2018-11-15

**Authors:** Pantelis Theodoros Nikolaidis, Bruno Matos, Filipe Manuel Clemente, Pedro Bezerra, Miguel Camões, Thomas Rosemann, Beat Knechtle

**Affiliations:** ^1^Exercise Physiology Laboratory, Nikaia, Greece; ^2^School of Sport and Leisure, Polytechnic Institute of Viana do Castelo, Melgaço, Portugal; ^3^Instituto de Telecomunicações, Delegação da Covilhã, Covilhã, Portugal; ^4^The Research Center in Sports Sciences, Health and Human Development, CIDESD, Vila Real, Portugal; ^5^Institute of Primary Care, University of Zurich, Zurich, Switzerland; ^6^Medbase St. Gallen Am Vadianplatz, St. Gallen, Switzerland

**Keywords:** cycle ergometer, field testing, football, jumping ability, performance, short-term muscle power, sprint

## Abstract

The Wingate anaerobic test (WAnT) has been used extensively to evaluate performance in soccer, however, a comprehensive sport-specific normative database has not been available so far. Therefore, the main aim of the present study was to develop norms of the main indices of the WAnT with regards to age in soccer. A secondary aim was to examine the relationship of WAnT with two common field tests, 20 m sprint and vertical jump, and study the variation of this relationship by age and playing position. Hundred and ninety five male soccer players (age 18.1 ± 4.9 years) performed the WAnT, and a sub-sample of 190 soccer players (age 19.4 ± 5.1 years) performed 20 m sprint, squat (SJ) and countermovement jump (CMJ). Age was related very largely with peak power (*R*^2^ = 0.57) and mean power of the WAnT (*R*^2^ = 0.60) when they were expressed in W, and largely (*R*^2^ = 0.41 and *R^2^* = 0.33, respectively) when they were expressed in W.kg^−1^, whereas it did not relate with fatigue index. After being adjusted for age, a relationship of SJ (*B* = 3.91, 90% CI: 2.49, 5.32; *R*^2^ = 0.26), CMJ (*B* = 3.59, 90% CI: 2.22, 4.95; *R*^2^ = 0.24) and 20 m sprint (*B* = −0.06, 90% CI: −0.10; −0.01; *R*^2^ = 0.19) with peak power of the WanT was observed. In summary, P_peak_ and P_mean_ were related very largely to age, especially during adolescence, and percentile norms of these indices were developed for 1-year age groups from 11 to 21 years old and for a single adult age group (22–39 years old). These findings on the largest dataset of soccer players ever studied would be expected to offer a practical tool to the members of the sports medicine team (e.g., exercise physiologists, fitness trainers, and coaches) working with them.

## Introduction

The Wingate anaerobic test (WAnT) has been a major laboratory test of short-term high-intensity performance in the field of soccer exercise physiology/exercise testing (Al-Hazzaa et al., 2001; Meckel et al., 2009). This test has been shown to differentiate soccer players from athletes of other sports (Kalinski et al., 2002; Harbili, 2015; Jakovljević et al., 2018). Also, it discriminated soccer players with cerebral palsy from their healthy peers and general population, too (Yanci et al., 2016). Moreover, it has been used as a golden standard to validate field tests in this sport (Thomas et al., 2002; Hazir et al., 2018) and it correlated with match performance (Pekas et al., 2016). It has been used to monitor the effectiveness of training, e.g., a 2 months preparation training program (Vasileios et al., 2018), 6 weeks training (Kazem et al., 2016) or 8 weeks training (Polczyk and Zatoń, 2015), and nutrition intervention in soccer players (Yáñez-Silva et al., 2017). Despite the documented popularity of the WAnT in soccer, surprisingly no comprehensive sport-specific normative data have been ever published. To the best of our knowledge the largest existing dataset of WAnT included 457 male athletes of various sports including soccer players, age 18–25 years old (Zupan et al., 2009), however, this dataset did not present classification of performance by sport.

On the other hand, short-term high-intensity performance in soccer might be evaluated out of the laboratory using the so-called field exercise tests. In the field, sprints and jumping tests would be selected based on their relevance with dynamic actions with increasing demands of muscle power and strength (Chamari et al., 2004). A popular running test in soccer was 20 m sprint and jump tests included squat (SJ) and countermovement jump (CMJ) (Loturco et al., 2017; Pojskic et al., 2018). It has been previously observed that sprint and jump tests were correlated with the WAnT in soccer (Nikolaidis et al., 2015, 2016). Furthermore, performance in the abovementioned exercise tests might vary by age (Asano et al., 2013) and playing position (Joo and Seo, 2016). Nevertheless, no information about the variation of the relationship of the WAnT with 20 m sprint, SJ and CMJ by age and playing position has been available so far. Soccer players usually were assigned into four playing positions (forward, midfielders, defenders, and goalkeepers) differing for their physiological demands (Gil et al., 2007; Deprez et al., 2013). In addition, soccer players were classified into age groups during the adolescence, considering their growth and maturation (Deprez et al., 2013). Therefore, the main aim of the present study was to develop norms of the main indices of the WAnT with regards to age in soccer. A secondary aim was to examine the relationship of WAnT with two common field tests, 20 m sprint and vertical jump, and study the variation of this relationship by age and playing position.

## Materials and Methods

### Study Design and Participants

A group of 995 soccer players from the region of Athens (age 18.1 ± 4.9 years; Table 1) voluntarily participated in the study and performed the WAnT. A sub-group of 190 participants (age 19.4 ± 5.1 years) performed also 20 m sprint, SJ and CMJ. The participants were from different soccer clubs from the region of Athens, where they practiced for 4–5 training sessions, each lasting ∼90 min, and participated in one soccer match per week. These clubs competed in the third and fourth national leagues of Greece. The testing procedures were performed during the preparation period of competitive seasons during 2009–2018. Eligibility criteria for this study were that the participants would be free of injury or illness during the research analyses. Prior to exercise testing, they had been instructed to maintain their physical activity and nutrition routines similar to those they used before matches. The institutional review board of Exercise Physiology Laboratory, Nikaia, Greece, approved this study and all participants provided their written informed consent. The experiment followed the ethical guidelines for the study of humans as suggested by the Declaration of Helsinki. Participants were grouped into four playing positions (goalkeepers, defenders, midfielders, and forward). In addition, the total sample was classified into 1 year age groups from 11 to 35 years to study the relationship of the WAnT with age and develop percentile norms. A sub-sample was divided into four age groups (12–14, 14–16, 16–18, and > 18 years old) to examine the combined effect of age and playing position on the relationship of the WAnT with 20 m sprint, SJ and CMJ.

**Table 1:** Anthropometric characteristics of participants.

	N	Age (years)	Weight (kg)	Height (cm)	BMI (kg/m^2^)	BF (%)
Total sample	995	18.1 ± 4.9	66.9 ± 12.0	173.3 ± 9.6	22.1 ± 2.6	15.6 ± 4.1
Sub-sample	192	19.4 ± 5.1	69.2 ± 10.8	175.8 ± 8.0	22.3 ± 2.2	14.1 ± 3.8

*BMI, body mass index; BF, body fat.*

### Procedures and Protocols

Each participant of the total group was tested once (WAnT), whereas the participants from the subgroup were tested during two sessions within a week and not on consecutive days (48 h interval). The first testing session took place in the laboratory, where they were examined for anthropometric characteristics (body height, body weight and skinfold thickness), and performed, SJ, CMJ, and the WAnT. During the second testing session, they were tested on a 20 m sprint in the field. The warm-up included a 10 min submaximal aerobic exercise and 10 min dynamic stretching exercises. Similar procedures of warm-up before sprinting and high-intensity tests have been used recently (Mann et al., 2015). This submaximal exercise was performed on a cycle ergometer in the first session and jogging in the second session.

Body height and weight were measured before warm-up during the first session using a stadiometer (SECA, Leicester, United Kingdom) and an electronic scale (HD-351 Tanita, IL, United States), respectively. Body Fat was estimated by skinfold thickness (Harpenden, West Sussex, United Kingdom) at 10 sites (cheek, wattle, chest I, triceps, sub- scapular, abdominal, chest II, suprailiac, thigh and calf; BF = −41.54 + 12.636 × log_e_x, where x was the sum of 10 skinfolds) using the Parizkova formula (Eston and Reilly, 2001). The skinfold thickness was calculated by a researcher with more than 10 years of experience in this procedure. Chronological age was calculated using a table of decimals of year. All anthropometric measurements were performed according to standardized procedures (Eston and Reilly, 2001).

In the two single vertical jump tests (SJ and CMJ), participants were asked to jump as high as possible (Aragon-Vargas, 2000) over a photocell platform (Opto-jump, Microgate Engineering, Bolzano, Italy). The two tests were performed on a randomized order. Two trials were performed for each jump test and the best one was recorded for further analysis. The height of each jump was calculated from the flight time. The participants were instructed beforehand in order to guarantee the proper jump technique.

The WAnT was performed on a cycle ergometer (Ergomedics 874, Monark, Sweden) (Driss and Vandewalle, 2013). Participants were instructed to pedal as fast as possible for 30 s against a braking force that was determined by the product of body mass in kg by 0.075. The following three main indices of the WAnT were evaluated: (a) peak power (Ppeak), (b) mean power (Pmean), and (c) fatigue index (FI). Both Ppeak and Pmean were expressed in W and W ⋅ kg^−1^. During this test, participants were encouraged verbally to exert maximal effort. Heart rate response to the WAnT was monitored by Team Pro (Polar Electro Oy, Kempele, Finland). The experiments in the laboratory were done in a temperature of 21°C.

The 20 m sprint test was performed at an outdoor soccer synthetic field (Nikolaidis et al., 2016). The test was administered twice and the best trial was recorded for further analysis. Each trial, starting from a standing position with the front foot placed 0.5 m before the first pair of photocells, was timed using three pairs of electronic timing gates (Brower Timing System, Salt Lake City, UT, United States) placed at 0, 10, and 20 m, as well as 1 m above the ground.

### Statistical Procedures

Cohen’s D (*d*) was used to evaluate the effect size test for differences between pairwise comparisons. The following classification of magnitude of *d* was applied (Ferguson, 2009): no effect (*d* < 0.41), minimum effect (0.41 < *d* < 1.15), moderate effect (1.15 < *d* < 2.70) and strong effect (*d* > 2.70). The partial eta squared (η^2^*p*) tested the effect size (ES). Ferguson’s classification for the ES was used (Ferguson, 2009): no effect (ES < 0.04); minimum effect (0.04 < ES < 0.25); moderate effect (0.25 < ES < 0.64); and strong effect (ES > 0.64). A generalized linear model was created to test the independent associations between the WAnT (above the mean) and the performance variables of the 20 m Sprint Test (speed), SJ (strength), and CMJ (strength). We computed beta coefficients with 90% confidence intervals (CI) were computed and coefficient of determination (*R*^2^) to describe the amount of variance/prediction on the dependent variable (speed and strength) among athletes after adjustment for age. All statistical analyses were performed with IBM SPSS version 19 and significance was set at *p* = 0.05.

## Results

**FIGURE 1:**
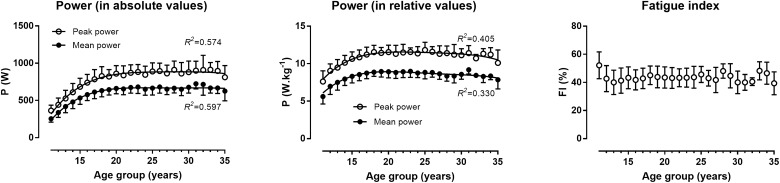
Peak and mean power in the Wingate anaerobic test expressed in absolute (left) or relative to body mass values (center), and fatigue index (right) by age group. Error bars represent standard deviations.

In the total sample, age was related very largely with peak power (*R*^2^ = 0.57) and mean power of the WAnT (*R*^2^ = 0.60) when they were expressed in W, and largely (*R*^2^ = 0.41 and *R*^2^ = 0.33, respectively) when they were expressed in W.kg^−1^, whereas it did not relate with fatigue index (Figure 1). The anthropometric characteristics were shown in Table 2. Normative data of WAnT indices were presented in Table 3.

**Table 2:** Anthropometric characteristics by age (*n* = 995).

	Age (years)												
	≤ 12	12	13	14	15	16	17	18	19	20	21	≥ 22
N	18	44	89	117	130	113	100	40	52	53	28	211
Age (years)	10.6 ± 1.4	12.6 ± 0.3	13.5 ± 0.3	14.5 ± 0.3	15.5 ± 0.3	16.5 ± 0.3	17.4 ± 0.3	18.4 ± 0.2	19.5 ± 0.3	20.5 ± 0.3	21.4 ± 0.3	27.1 ± .7
Weight (kg)	43.3 ± 10.9	49.0 ± 9.0	55.5 ± 9.1	60.1 ± 9.5	64.3 ± 8.5	68.3 ± 9.5	69.8 ± 8.9	71.4 ± 7.0	72.1 ± 6.9	74.4 ± 7.2	74.1 ± 8.8	76.8 ± 8.2
Height (cm)	144.3 ± 8.4	156.1 ± 8.0	165.6 ± 7.2	169.2 ± 8.2	173.1 ± 6.3	176.0 ± 6.4	176.5 ± 6.4	177.3 ± 5.6	176.9 ± 5.4	177.7 ± 5.8	177.9 ± 8.0	179.1 ± 6.3
BMI (kg.m^−2^)	20.6 ± 3.7	20.0 ± 2.4	20.2 ± 2.4	20.9 ± 2.5	21.4 ± 2.3	22.0 ± 2.7	22.4 ± 2.2	22.7 ± 1.7	23.0 ± 1.8	23.5 ± 1.8	23.4 ± 2.0	23.9 ± 2.0
BF (%)	19.6 ± 6.6	17.0 ± 5.4	15.8 ± 4.4	16.3 ± 4.6	15.4 ± 3.8	15.0 ± 3.7	15.1 ± 3.5	13.2 ± 3.2	15.2 ± 3.5	14.7 ± 3.3	14.6 ± 3.7	16.0 ± 3.8
FM (kg)	9.0 ± 5.4	8.7 ± 4.1	9.0 ± 3.7	10.0 ± 3.9	10.1 ± 3.5	10.5 ± 4.1	10.7 ± 3.6	9.4 ± 2.5	11.0 ± 3.1	11.0 ± 3.0	11.0 ± 3.9	12.4 ± 3.9
FFM (kg)	34.2 ± 6.0	40.4 ± 6.0	46.6 ± 6.4	50.1 ± 6.9	54.2 ± 6.1	57.8 ± 6.1	59.2 ± 6.5	61.9 ± 6.5	61.0 ± 5.2	63.4 ± 5.8	63.0 ± 5.9	64.4 ± 5.8

*BMI, body mass index; BF, body fat; FM, fat mass; FFM, fat-free mass.*

**Table 3:** Percentiles of the main indices of the Wingate anaerobic test by age.

	Age (years)											
Percentile	12	13	14	15	16	17	18	19	20	21	≥ 22
**Peak power (W)**											

95	645	694	833	887	908	1005	1078	969	1034	1028	1056
90	570	671	778	818	893	917	1037	928	968	1021	1023
75	505	611	671	764	826	859	890	899	934	933	954
50	425	540	610	680	732	787	809	829	857	854	882
25	379	433	536	601	668	710	752	748	774	732	776
10	330	380	452	538	586	603	660	684	737	649	723
5	319	342	410	490	549	552	639	659	699	601	677

**Peak power (W.kg^−1^)**											

95	10.5	11.1	11.9	12.2	12.4	12.8	13.3	12.5	13.0	13.0	12.9
90	10.3	10.9	11.5	11.9	12.1	12.5	12.7	12.4	12.6	12.8	12.6
75	9.8	10.2	10.7	11.2	11.5	11.9	12.0	11.9	12.2	12.3	12.0
50	9.1	9.5	10.1	10.6	10.9	11.2	11.5	11.5	11.5	11.5	11.5
25	8.4	8.7	9.5	10.1	10.4	10.4	10.9	11.0	11.0	11.0	10.8
10	7.7	8.1	8.9	9.5	9.6	9.8	10.6	10.6	10.3	9.9	9.9
5	7.2	7.3	8.6	9.2	9.2	9.5	10.0	10.3	9.8	9.8	9.7

**Mean power (W)**											

95	455	539	658	669	713	769	811	764	785	773	785
90	440	529	588	642	681	710	734	743	761	760	765
75	394	479	534	585	632	671	683	699	700	723	722
50	323	428	478	533	580	620	616	648	659	657	673
25	287	352	420	474	527	562	572	592	618	577	615
10	261	299	359	426	477	499	540	541	586	537	556
5	249	284	332	375	431	455	512	530	546	516	529

**Mean power (W.kg^−1^)**											

95	8.4	9.1	9.3	9.7	9.7	9.9	10.0	10.1	10.2	9.9	9.8
90	8.1	8.8	9.0	9.4	9.5	9.7	9.7	9.9	10.0	9.7	9.6
75	7.9	8.2	8.5	8.9	9.1	9.3	9.2	9.5	9.4	9.4	9.2
50	7.2	7.6	7.9	8.4	8.6	8.8	8.9	9.0	9.0	8.9	8.8
25	6.3	6.9	7.5	7.7	8.2	8.3	8.5	8.6	8.3	8.5	8.3
10	5.3	5.9	6.7	7.1	7.5	7.8	8.1	8.1	8.0	7.7	7.8
5	4.9	5.6	6.3	6.7	7.2	7.3	7.8	7.5	7.7	6.7	7.1

**Fatigue index (%)**											

95	58.3	56.1	56.3	55.9	51.8	55.1	55.5	64.3	57.8	55.0	54.8
90	56.5	52.6	52.8	52.6	49.3	53.0	53.3	52.5	53.7	52.9	52.5
75	49.6	45.6	45.3	47.8	46.2	47.6	49.8	46.9	47.3	47.9	48.7
50	40.5	39.2	41.0	43.2	41.7	43.1	46.0	42.4	43.8	45.1	44.2
25	35.7	34.2	35.8	38.1	37.5	37.7	41.8	38.7	39.5	38.4	39.1
10	29.9	29.1	30.7	33.0	32.6	34.6	38.7	35.8	33.7	32.1	33.3
5	29.1	26.2	26.0	31.9	31.2	31.2	28.3	33.5	29.3	29.5	29.3

With regards to the sub-group that performed WAnT, 20 m sprint, SJ, and CMJ, descriptive statistics by age group and playing position were shown in Table 4. The analysis of age × age group indicated interactions for Ppeak (*p* = 0.316; η^2^*p* = 0.057, minimum effect), Pmean (*p* = 0.147; η^2^*p* = 0.072, minimum effect) and CMJ (*p* = 0.073; η^2^*p* = 0.084, minimum effect), 20 m (*p* = 0.016; η^2^*p* = 0.107, minimum effect), and SJ (*p* = 0.011; η^2^*p* = 0.112, minimum effect).

**Table 4:** Mean and [90% Confidence Interval] for performance variables split by age group and playing positions.

	SJ (cm)	CMJ (cm)	20 m (s)	Ppeak (W)	Pmean (W)
**12–14 YO (*N* = 16)**					
GK (*N* = 1)	32.5	32.1	3.41	341	309
DF (*N* = 4)	26.5 [22.6;30.4]	29.2 [25.1;33.3]	3.41 [3.29;3.53]	559 [446;673]	448 [364;532]
MF (*N* = 7)	27.3 [22.1;32.4]	30.2 [25.8;34.6]	3.40 [3.27;3.53]	533.43 [439;627]	435 [362;509]
FW (*N* = 4)	23.6 [20.1;27.1]	23.8 [20.7;27.0]	3.57 [3.39;3.75]	516 [453;578]	385 [317;454]
**14–16 YO (*N* = 36)**					
GK (*N* = 3)	28.8 [22.3;35.4]	31.4 [27.8;34.9]	3.37 [2.93;3.81]	693 [402;983]	551 [352;751]
DF (*N* = 11)	26.9 [24.1;29.6]	29.1 [26.4;31.8]	3.24 [3.15;3.33]	668 [578;758]	506 [448;563]
MF (*N* = 16)	28.6 [26.5;30.6]	30.0 [28.3;31.7]	3.19 [3.13;3.24]	653 [624;682]	510 [489;531]
FW (*N* = 6)	24.2 [21.7;26.8]	26.7 [23.7;28.9]	3.39 [3.23;3.54]	569 [447;691]	425 [350;499]
**16–18 YO (*N* = 47)**					
GK (*N* = 4)	27.3 [22.9;31.7]	30.0 [25.1;34.8]	3.29 [3.17;3.40]	788 [556;1020]	593 [453;733]
DF (*N* = 21)	29.0 [27.0;31.0]	31.8 [29.8;33.8]	3.20 [3.15;3.25]	754 [714;795]	578 [543;613]
MF (*N* = 16)	32.3 [29.4;35.3]	33.9 [31.2;36.6]	3.13 [3.07;3.19]	745 [699;790]	588 [555;620]
FW (*N* = 6)	36.6 [32.5;40.7]	36.7 [33.6;39.8]	3.09 [2.98;3.19]	831 [662;1000]	646 [517;774]
**>18 YO (*N* = 93)**					
GK (*N* = 7)	36.0 [33.6;38.3]	37.3 [34.9;39.6]	3.10 [3.03;3.17]	948 [888;1007]	698 [660;736]
DF (*N* = 33)	36.0 [34.7;37.2]	37.7 [36.4;39.0]	3.08 [3.05;3.10]	900 [864;936]	684 [658;711]
MF (*N* = 41)	35.3 [34.2;36.4]	37.4 [36.2;38.6]	3.07 [3.05;3.10]	852 [821;883]	650 [628;671]
FW (*N* = 12)	34.7 [32.6;36.7]	36.0 [34.2;37.9]	3.07 [3.02;3.11]	895 [813;977]	682 [626;739]

*GK, goalkeeper; DF, defender; MF, midfielder; FW, forward; yo, years old; SJ, squat jump; CMJ, counter-movement jump; Ppeak, peak power; Pmean, mean power.*

### Inter-Age Group Changes

Moderate differences among age groups were found on Ppeak (*p* = 0.001; η^2^ = 0.475), Pmean (*p* = 0.001; η^2^ = 0.477), 20 m (*p* = 0.001; η^2^ = 0.431), SJ (*p* = 0.001; η^2^ = 0.349), and CMJ (*p* = 0.023; η^2^ = 0.370) with older age groups outscoring their younger counterparts. The 12–14 group had lower Ppeak than the 14–16 group (*d* = 1.022, minimum effect), 16–18 group (*d* = 1.935, moderate effect) and >18 group (*d* = 2.924, strong effect); lower Pmean than the 14–16 (*d* = 0.936, minimum effect), 16–18 group (*d* = 1.824, moderate effect) and > 18 group (*d* = 2.917, strong effect); slower 20 m sprint than the 14–16 group (*d* = 1.138, minimum effect), 16–18 group (*d* = 1.933, moderate effect) and > 18 group (*d* = 3.640, strong effect); lower SJ than the 16–18 group (*d* = 0.750, minimum effect) and > 18 group (*d* = 2.081, moderate effect); and lower CMJ than the 16–18 group (*d* = 0.844, minimum effect) and > 18 group (*d* = 2.033, moderate effect).

The 14–16 age group had lower Ppeak than the 16–18 group (*d* = 0.899, minimum effect) and >18 group (*d* = 1.870, moderate effect); lower Pmean than the 16–18 group (*d* = 0.993, minimum effect) and > 18 group (*d* = 1.994, moderate effect); slower 20 m sprint than the 16–18 group (*d* = 0.543, minimum effect) and >18 group (*d* = 1.517, moderate effect); lower SJ than the 16–18 group (*d* = 0.649, minimum effect) and >18 group (*d* = 1.898, moderate effect); lower CMJ than the 16–18 group (*d* = 0.766, minimum effect) and >18 group (*d* = 1.929, moderate effect). The 16–18 age group had lower Ppeak than the >18 group (*d* = 0.954, minimum effect); lower Pmean than the > 18 group (*d* = 0.895, minimum effect); slower 20 m than the > 18 group (*d* = 0.893, minimum effect); lower SJ than the > 18 group (*d* = 0.924, minimum effect); and lower CMJ than the >18 group (*d* = 0.922, minimum effect).

### Between-Playing Positions Changes

No effect between playing positions was found in Ppeak (*p* = 0.325; η^2^ = 0.017), Pmean (*p* = 0.545; η^2^ = 0.010), 20 m (*p* = 0.078; η^2^ = 0.033), SJ (*p* = 0.653; η^2^ = 0.008,) and CMJ (*p* = 0.344; η^2^ = 0.016).

### Prediction of WAnT From 20 m Sprint and Jump Tests

The modeling of the associations of the WAnT with speed (20 m sprint) and strength (SJ and CMJ) can be seen in Table 5. After being adjusted for age, a relationship of SJ, CMJ, and 20 m sprint with peak power of the WanT was observed.

**Table 5:** Modeling the relationship of the Wingate anaerobic test with the performance variables of speed (20 m sprint) and strength (squat and countermovement jump) after adjustment for age.

Parameter	Beta (*B*)	90% CI	*R*^2^
**Prediction of Speed (20 m Sprint Test as Dependent Variable, s)**			
Above the mean on Wingate test (778 W)	−0.06	−0.10; −0.01	0.19
**Prediction of Strength (SJ as Dependent Variable, cm)**			
Above the mean on Wingate test (778 W)	3.91	2.49; 5.32	0.26
**Prediction of Strength (CMJ as Dependent Variable, cm)**			
Above the mean on Wingate test (778 W)	3.59	2.22; 4.95	0.24

*CI, confidence intervals; R^2^, coefficient of determination.*

## Discussion

The main findings of the present study were that (a) age was related very largely with peak power and mean power of the WAnT, whereas it did not relate with fatigue index, (b) since the WAnT indices varied by age, especially during adolescence, percentile norms were developed for 1 year age groups, (c) a main effect of age on the WAnT, 20 m sprint, SJ, and CMJ was observed with the older the age group, the better the performance, and (d) no difference in testing outcomes by playing position was shown.

The differences in 20 m sprint, SJ and CMJ showed similar trend, i.e., the older age groups had better outcome in all performances than the younger age groups. Moreover, it should be highlighted that these differences tended to decrease across adolescence, an observation which was in agreement with previous research (Slimani and Nikolaidis, 2017). Regarding 20 m sprint, the results were in line with previous research (Al Haddad et al., 2015), which found better performance by adults in comparison to adolescent soccer players, while older adolescents performed better than younger adolescents, and no differences were observed between the adult groups. The findings in Ppeak and Pmean of the WAnT confirmed previous research, where differences were shown among age groups across adolescence, with the age groups in the higher spectrum of adolescence performing better than those in the lower spectrum (Slimani and Nikolaidis, 2017). A novel finding was that no differences in Ppeak and Pmean were observed among adult soccer players indicating that long-term adaptations to soccer training might be adequate for the maintenance of performance in the WAnT.

The increase of Ppeak and Pmean across adolescence coincided with an increase of weight and FFM during this period of human life. This finding was in agreement with Carvalho et al. (2011) who observed that the increments in the WAnT of athletes were related to both corresponding increments of weight and FFM during this period. Very large correlations of FFM with Ppeak and Pmean have been shown in another study (Kim et al., 2011), which would suggest that a lack of differences in Ppeak and Pmean among age groups of adult soccer players might be due to their similar weight and FFM.

Considering that the WAnT was the gold standard in the laboratory assessment of anaerobic power in athletes (Zupan et al., 2009), it was examined whether the WAnT could predict the values of performance variables such as sprint and vertical jump. Since adolescence had an important contribution in the development of strength and power (Deprez et al., 2013; Lovell et al., 2015), the values were adjusted to the age. An association of Ppeak with 20 m sprint, SJ, and CMJ was observed in the present study, where Ppeak could independently explain a large proportion of 20 m sprint (19%) and jumping ability (24–26%).

### Limitations, Strength, and Practical Applications

A limitation of the present study was that it considered adult soccer players of a specific performance level (their clubs competed in the third and forth national division), whereas the adolescent soccer players could not be characterized for their performance level as they were not competed to national divisions. Thus, caution would be needed to generalize these findings in soccer players with different performance level. On the other hand, strength of the study was its novelty as it presented WAnT data of the largest sample of soccer players ever studied. The large sample offered the opportunity to examine differences among adult age groups and develop percentile norms for 1 year age groups across adolescence. Knowledge of the variation of performance indices, such as WAnT, 20 m, SJ, and CMJ by age, especially across adolescence, would be of great practical value for the members of the sports medicine team working with soccer players. These norms might be used for purposes of talent identification, soccer players’ selection, monitoring training and rehabilitation. Using norms 1 year age groups would be of particular value in adolescence, which was a period of human life with large changes in short-term high-intensity performance (Deprez et al., 2013).

## Conclusion

In summary, the main indices of the WAnT (Ppeak, Pmean, and FI) were examined in the large database of (∼1000) male soccer players from 11 to 39 years old. Since P_peak_ and P_mean_ were related very largely to age, especially during adolescence, percentile norms these indices were developed for 1 year age groups from 11 to 21 years old and for a single age group from 22 to 39 years old. Furthermore, we confirmed the moderate to large relationship of the WAnT with 20 m sprint, SJ, and CMJ in a sub-sample of (∼200) soccer players. These findings would be expected to fill a major gap in exercise testing of male soccer players and offer a practical assessment tool to the members of the sports medicine team (e.g., exercise physiologists, fitness trainers, and coaches) working with them.

## Author Contributions

PN and BM conceived the study. PN, FC, PB, and MC designed the study. PN collected data. PN and BM analyzed and interpreted the data and drafted the manuscript. BM, FC, PN, PB, MC, TR, and BK revised the manuscript and approved the final version.

## Conflict of Interest Statement

The authors declare that the research was conducted in the absence of any commercial or financial relationships that could be construed as a potential conflict of interest.
